# iPLA2β-null mice exposed to natural pathogens exhibit hepatocellular fibrotic injury with male-biased alteration of glycerolipid metabolism

**DOI:** 10.3389/fimmu.2026.1839554

**Published:** 2026-07-08

**Authors:** Weihong Xu, Jiliang Wang, Gang Li, Gerhard Liebisch, Walee Chamulitrat

**Affiliations:** 1 Department of Rheumatology, the Second Affiliated Hospital of Zhejiang University School of Medicine, Hangzhou, China; 2 Department of Gastrointestinal Surgery, Union Hospital, Tongji Medical College, Huazhong University of Science and Technology, Wuhan, China; 3 Institute of Clinical Chemistry and Laboratory Medicine, University of Regensburg, Regensburg, Germany; 4 Department of Medicine IV, Gastroenterology, Hepatology and Infectious Diseases, Heidelberg University, Heidelberg, Germany

**Keywords:** chronic liver disease, fatty acids, lipoprotein secretion, liver lipid synthesis, phospholipid metabolism, PLA2G6, whole-body knockout mice

## Abstract

Group VIA calcium-independent phospholipaseA2 (iPLA2β or PLA2G6) is a homeostatic enzyme involved in basal glycerophospholipid metabolism. The mutations in the *PLA2G6* gene lead to heterogenous neurodegenerative disorders. Global *PLA2G6* inactivation in iPLA2β-null mice exhibited liver fibrosis and intestinal atrophy when they reached an advanced age at 20–22 months old. Here, we analyzed the phenotypes of iPLA2β-null mice which happened to be exposed to natural pathogens in our animal facility. Compared with wild-type, male iPLA2β-null mice at 9–14 months of age exhibited reduced body, liver, and subcutaneous fat weights concomitant with decreased hepatic triacylglycerol and decreased expression of *de novo* lipogenesis genes. Hepatocytes from male mutants were sensitive to apoptosis induced by palmitic acid. Male but not female mutants displayed attenuation of hepatic lipid synthesis; however hepatic fibrosis was increased in mutants of both sexes. Hepatic apoptosis was also increased in mutants of both sexes, and they were susceptible to endotoxin-induced liver injury. Hence, global *PLA2G6* inactivation combined with natural infection accelerates progression of chronic liver disease in both male and female mice with male-biased alteration of hepatocellular glycerolipid metabolism.

## 1 Introduction

The liver is the central regulatory organ of lipid metabolism and plays a role in the synthesis of lipoproteins. An alteration in hepatocellular lipid metabolism mediates inflammation and chronic liver disease (1). Glycerophospholipids (GPL) play a crucial role in homeostasis of the liver through membrane stabilization (2) acting as a source of triacylglycerol (TG) (3) and constituents of lipoproteins (4). In the regulation of GPL remodeling, phospholipases including phospholipase A2 (PLA2) are important for an establishment of membrane homeostasis. Among the known 15 mammalian PLA2 enzymes (5), the widely expressed group VIA calcium-independent phospholipase A2 (iPLA2β or PLA2G6) has been intensively studied in nearly three decades (6). This enzyme does not require calcium for the hydrolysis of GPL at the *sn*-2 position to generate a lysophospholipid and a fatty acid (FA). Given its central role in GPL remodeling, *PLA2G6* has been implicated in human metabolic traits. Indeed, *PLA2G6* is identified as one of the twelve loci that reached significance in genome-wide association study of body fat percentage over 100, 000 individuals of primarily European ancestry (7). The body fat percentage-increasing allele in the locus near *PLA2G6* is associated with lower TG (8). *PLA2G6* single-nucleotide polymorphisms (SNPs) in >100, 000 individuals are negatively associated with plasma TG (9). Finally, *PLA2G6* SNPs are also associated with serum C-reactive protein among non-European-ancestry populations (10) and in those individuals subjected to a 6-week supplementation with fish oil (11). Thus, the variability of *PLA2G6* is associated with adiposity, plasma TG, and inflammation. However, its functional role in liver disease remains unclear, particularly, in populations who consume regular diets.

The mutations in *PLA2G6* gene lead to heterogeneous neurodegenerative disorders so-called PLA2G6-associated neurodegeneration (PLAN) including infantile neuroaxonal dystrophy, atypical neuroaxonal dystrophy, and Parkinson’s syndrome (12). The overall prevalence of PLAN is 1 in 1 million, and the prevalence is the highest among African/African-Americans and East-Asians (13). Consistently, iPLA2β-null mice with global *PLA2G6* deficiency has served as a genetic model for progressive human motor disorders (14). In addition to brain, pathological abnormalities of iPLA2β-null mice have been reported in testis (15), pancreas (16), and bones (17). We also reported that male iPLA2β-null mice at an advanced age of 20–22 months old displayed liver fibrosis and intestinal atrophy (18). Activation of phagocytes and macrophages was observed in livers of iPLA2β-null mice during acute injury induced by concanavalin A (19) and anti-CD95/FasL antibody (20). In the latter case, Kupffer cells from male mutants displayed elevated release of pro-inflammatory cytokines (20). Hence, global *PLA2G6* deficiency elicits systemic effects associated with macrophage activation and abnormalities in several tissues.

Regarding liver metabolism, male iPLA2β-null mice at 6 (21) and 12 (22) months of age under chow feeding did not show any changes in the total lipid species of GPL subclasses, namely, phosphatidylcholine (PC), phosphatidylethanolamine (PE), phosphatidylserine (PS), and phosphatidylinositol (PI). However, they showed an elevation of individual lipid species including PC and PE containing arachidonic acid (AA) (21), and PE 34:2 (likely PE 16, 18:2) (22). This indicates the specificity of *PLA2G6* for AA and linoleic acid as reported in the brain (23). Even though the levels of hepatic FA were not altered in iPLA2β-null mice (21, 22), earlier studies in cultured cells demonstrated that iPLA2β inhibitors were able to suppress the synthesis of TG (24) and lipoprotein secretion (25). In this brief research report, we performed phenotyping of iPLA2β-null mice at middle age of 9–14 months old that happened to be exposed to natural pathogens in our animal facility. While not seen in female mutants, male mutants exhibited suppression of hepatic TG synthesis and lipoprotein secretion. However, mutants of both sexes exhibited exaggerated hepatic fibrosis, apoptosis and injury susceptibility upon exposure to endotoxin lipopolysaccharides (LPS). Thus, global *PLA2G6* deficiency induced sex-biased alteration of hepatocellular lipid synthesis and exacerbation of infection-induced hepatic inflammatory injury in both sexes.

## 2 Materials and methods

### 2.1 iPLA2β-null mice and treatment

iPLA2β-null mice were kind gifts from Dr. John Turk (Washington University School of Medicine, St. Louis, Missouri, USA). Mice were bred with C57BL/6 background and genotyped based on published work (18–22). Age-matched C57BL/6 mice in our in-house breeding colonies were used as WT controls. WT and mutant mice were housed in cages without filters together with mice from other research groups at the animal facility of Heidelberg University. Infection of laboratory mice with natural pathogens can occur in animal facility, and this could have effects on research results. The records from our animal facility (**Supplementary Material**) indicated the presence of mouse *norovirus* (detected by indirect immunofluoresecence) *Helicobacter* sp., and *Helicobacter hepaticus* (detected by polymerase chain reaction) at the time of our experiments in 2012-2013. This exposure may have led to natural infection and sensitization to injury in mutant mice in our publications in 2015 (19, 20). After 2015, all of our mice were housed in cleaned rooms, and these mice were used in 2017–2019 publications (18, 21, 22).

For hepatic lipid synthesis, male mice at 6 or 12 months of age (4 mice per group) were fasted overnight and intravenously injected with 500 mg/kg tyloxapol (#T8761, Sigma) via tail vein and immediately followed by an oral administration with 0.5 ml lipid emulsion containing 400 μl intralipids (#I141, Sigma) and 100 μl of corn oil (#C8267, Sigma). Tyloxapol is an inhibitor of lipoprotein lipase without side effects and is commonly used to study hepatic lipid synthesis *in vivo* (26). Blood was taken from tail vein prior to treatment and post treatment every hour for 5 h. For LPS cohort (4 mice per group), male and female mice at 4 and 12 months of age were intraperitoneally injected with saline or 1 mg/kg LPS (Sigma, Taufkirchen, Germany), and were fasted but with free-access to water for 16 h. Mice were euthanized upon exposure to CO_2_ (~2-6% of chamber volume per minute through a 2.5-mm orifice) in a closed 3-liter chamber, initially by low flow rates (~48 liters per min) to establish anesthesia and followed by high flow rates (~180 liters per min) to euthanize. Pinching mouse toes was tested to ensure complete euthanization. Blood, liver and subcutaneous fat were harvested, snap-frozen, and stored at -80°C. Studies involving animal experimentation were approved by Heidelberg University Institutional Animal Care and Use Committee, and the German Authority (Baden-Württemberg Regierungspräsidium Karlsruhe) with license number 35-9185.81/G248/11. All procedures were followed according to Animal Welfare Laboratory Animal Ordinance from the German Animal Welfare Act.

### 2.2 Hepatocyte preparation and treatment

For hepatocyte preparation, WT and iPLA2β-null mice at 2 months old were used. Mice were anesthesized by an intraperitoneal injection using a 1-ml syringe containing ketamine and xylazine to give a dosage of 30 mg/kg and 3 mg/kg, respectively. After ensuring complete anesthesia by pinching mouse toes, midline incision was performed for canula insertion to portal vein. Liver perfusion then was performed in two steps with first perfusion buffer (HBSS with phenol red containing 10 mM glucose, 2 mM lactic acid, 0.2 mM pyruvate, 21 mM NaHCO_3_, 0.8 mM MgCl_2_, 0.5 mM EGTA, and 2 mM L-glutamine) and subsequently with perfusion buffer containing 0.06g/100 ml collagenase type CLSII (Biochrom AG, Berlin, Germany). Mice were automatically euthanized by the procedure of liver perfusion. Perfused liver was teased by using sharp tweezers to release hepatocytes which were then collected. Hepatocytes were then purified by using a 58%-Percoll (GE Healthcare, Freiburg, Germany) gradient, and were allowed to adhere to collagen-coated plates in M199 medium (PAA, Cölbe, Germany) containing 5% calf serum, 100 nM dexamethasone, 0.5 nM insulin, and antibiotics for 4 h. After 4 h, medium was replaced with same medium without serum. Hepatocytes were either harvested 20 h later for analysis or treated with 300 μM palmitate (Pal) containing 0.5% BSA and 0.1% ethanol or (BSA/ethanol) control for 20 h. The procedure for hepatocyte preparation was approved by Animal Care and Use Committee of Heidelberg University and the German Authority (Baden-Württemberg Regierungspräsidium Karlsruhe) with a license number 35-9185.81/G248/11.

### 2.3 Lipid extraction and analyses

Mouse hepatocytes were lysed with 100 μl PBS containing 1% TritonX-100. For livers and subcutaneous fat, 100 mg tissues were homogenized in 1 ml homogenizing buffer (50 mM Tris.HCl, pH 7.4, 1 mM EDTA, 10 mM NaF, and 1 mM DTT, and protease inhibitor cocktails). Hepatocyte lysates and tissues homogenates of 50 mg protein adjusted with PBS to 1 ml were used for lipid extraction using 4 ml 2:1 chloroform:methanol. After vortexing and centrifugation, supernatants were transferred to a new tube, and 1 ml of 50 mM citric acid, 2 ml deionized water, and 1 ml chloroform was added and well mixed. After centrifugation, the lower chloroform phase was separated and evaporated to complete dryness. Lipids were dissolved in 100 μl 3:2 hexane:isopropanol and kept in -20°C until lipid analyses. TG and non-esterified free fatty acids (NEFA) were determined by enzymatic kits using LabASSY TG and NEFA-HR kits (Wako Chemicals GmbH, Neuss, Germany), respectively. Cholesterol (Chol) and GPL were analyzed by using kits from Randox (Krefeld, Germany) and MIT diagnostics (Idstein, Germany) respectively.

### 2.4 Analyses of plasma samples

Plasma samples were prepared by centrifugation of blood at 2500 rpm at 4°C for 5 min. Plasma TG levels were determined by enzymatic kits using LabASSY TG kits (Wako Chemicals GmbH, Neuss, Germany). Plasma activities of lactate dehydrogenase (LDH) and aspartate aminotransferase (AST) were determined using Randox kits (Krefeld, Germany). Plasma caspase3/7 activities were determined using Caspase 3/7^Glo^ assay kits (#G8090, Promega, Mannheim, Germany), and luminescence was measured with a Fluostars Optima (BMG Labtech GmbH, Germany). Lipoprotein profiles in mouse plasma were analyzed by using an on-line dual-enzymatic method with gel-permeation high-performance liquid chromatography at Liposearch Skylight Biotech, Akita, Japan (27). This method measured TG and Chol levels in each lipoprotein fraction: chylomicrons (CM, >80 nm), very-low-density lipoproteins (VLDL, 30–80 nm), low-density lipoproteins (LDL, 16–30 nm), and high-density lipoproteins (HDL, 8–16 nm).

### 2.5 Lipidomic analyses

For liver samples of 12-months old mice post tyloxapol and lipid treatment, the lipidomic profiling of FA was analyzed by gas-chromatography mass spectrometry (GC-MS) (28), and quantification of GPL subclasses was analyzed by a direct flow injection electrospray-ionization mass spectrometry (ESI/MS-MS) in positive ion mode as previously described (29).

### 2.6 Gene expression

RNeasy Mini Kit (Qiagen, Hilden, Germany) were used to prepare RNA from liver. RNA was reversed transcribed using Maxima First Strand cDNA synthesis kit (Thermo Scientific, St. Leon-Rot, Germany). Quantitative real-time polymerase-chain-reaction (qRT-PCR) was performed on an Applied Biosystems 7500 System (Thermo Fisher Scientific) using TaqMan^®^ Gene expression assays (30). The comparative Ct (ΔΔCt) method was used to determine gene expression of a target normalized to the housekeeping gene GAPDH.

### 2.7 Western blotting

Proteins from liver or hepatocyte homogenates (30 μg proteins) and plasma samples (5 μl) were separated by SDS-PAGE, transferred onto a PVDF membrane, and blocked in 5% milk. Membranes were incubated with a primary antibody against FASN (#3180, Cell Signalling), ELOVL6 (ab69857, Abcam), cleaved PARP-1 (cleaved p25, 1051-1, Epitomics), PUMA (#4976, Cell Signalling), CHOP (sc-7351, Santa Cruz), ApoB (s-18, sc-11795, Santa Cruz), cleaved caspase 3 (#9664, Cell Signaling), Bax (#2774, Cell Signalng), Bcl2 (#2876, Cell Signaling), Bcl-xL (#2762, Cell Signaling), MCP-1 (#2027, Cell Signaling), and GAPDH (#2118, Cell Signaling). Following an incubation with a goat anti-mouse (sc-2005, Santa Cruz) or anti-rabbit (#7074, Cell Signaling) HRP-linked secondary antibody, proteins were visualized using Luminata Forte ECL (Millipore, Darmstadt, Germany). Image J software (http://imagej.nih.gov/ij/) was use to analyze band density for quantification of ApoB per 5 μl plasma and cleaved Parp-1/Gapdh ratio.

### 2.8 Histology and immunohistochemistry

Paraffin blocks from formalin-fixed livers were sectioned to 4-μm thick. For histology, slides were stained with hematoxylin and eosin (H&E). Sirius Red was used to stain for fibrotic collagens according to standard protocols. For IHC procedure, antigen retrieval of samples was performed by heating in citrate buffer, pH 6, for 20 min. After blocking endogenous peroxidase activity with hydrogen peroxide, followed by serum blocking, slides were incubated overnight with a primary antibody against α-Sma (1:250, E184, Epitomics/Biomol), F4/80 (1:100, ab11110, Abcam), and CK-19 (1:100, ab133496, Abcam). Goat anti-rabbit HRP-linked secondary antibody (ab6721, Abcam) and diaminobenzidine were used for detection. Apoptosis TUNEL-staining of liver samples was performed using DeadEnd™ Colorimetric TUNEL System (# G7130, Promega, Mannheim, Germany) according to company’s instructions. Light microscopy was used to visualize stained slides with an Olympus AX 70 microscope.

### 2.9 Statistical analysis

The statistical analysis was performed by using GraphPad Prism 5 (GraphPad, La Jolla, CA, USA). Data were expressed as mean ± SD. Statistical significance analyses were analyzed by two-tailed Mann-Whitney U tests for two-group comparisons. P < 0.05 was considered statistically significant.

## 3 Results

### 3.1 Male iPLA2β-null mice exposed to natural pathogens exhibited reduction in adiposity and hepatic lipids, and susceptibility to palmitate-induced apoptosis *in vitro*

**Figure 1:**
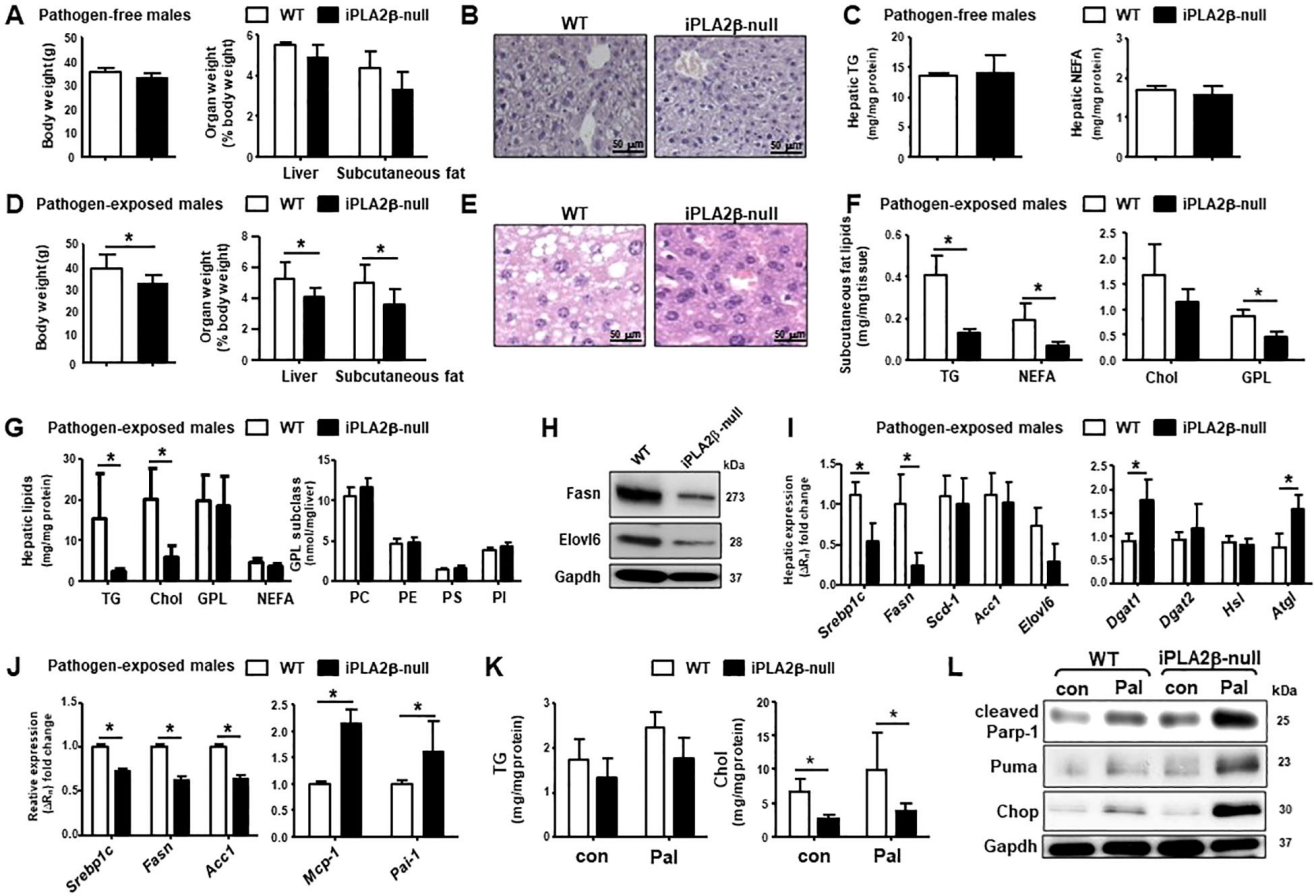
Male iPLA2β-null mice exposed to natural pathogens exhibited reduction in adiposity and hepatic lipids, and susceptibility to palmitate-induced apoptosis *in vitro*. Pathogen-free and pathogen-exposed male iPLA2β-null and C57BL/6 (WT) mice at 9–14 months of age were analyzed. Hepatocytes were prepared from pathogen-exposed male mice at 2 months of age. For pathogen-free cohort, **(A)** body weights (g) and the percentage of liver and subcutaneous fat per body weights (N = 5-6); **(B)** representative H&E staining of livers; and **(C)** the levels of hepatic TG and NEFA (N = 5-6). For pathogen-exposed cohort, **(D)** body weights (g) and the percentage of liver and subcutaneous fat per body weights (N = 9-12); **(E)** representative H&E staining of livers; **(F)** the levels of subcutaneous TG, NEFA, Chol, and GPL (N = 4-6); **(G)** the levels of hepatic TG, Chol, GPL, and NEFA as determined by enzymatic kits as well as hepatic GPL subclass: PC, PE, PS, and PI as determined by ESI/MS-MS (N = 4-6); **(H)** hepatic protein expression of Fasn and Elovl6; and **(I)** hepatic mRNA expression of *Srebp1c, Fasn, Scd-1, Acc1*, *Elovl6*, *Dgat1*, *Dgat2*, *Hsl*, and *Atgl* (N = 4-6). For hepatocytes isolated from pathogen-exposed mice, **(J)** expression of *Srebp1c, Fas, Acc1, Mcp-1, and Pai-1* (N = 3–5 mice per group); **(K)** cellular TG and Chol levels were determined in hepatocytes treated with control or 300 μM palmitate (Pal) for 20 h (N = 6 wells per group); and **(L)** hepatic protein expression of cleaved Parp-1, Puma, and Chop in hepatocytes treated with control or Pal for 20 h. Data are mean ± SD. *p < 0.05 by two-tailed Mann Whitney U tests.

In 2011, we expanded mouse colonies from two 2 heterozygous mice obtained from Dr. Turk. Because of many breeding rounds of small colonies requiring extended time together with the pregnancies of female mice, we obtained a cohort of male control and iPLA2β-null mice at 9–14 months of age for our first studies. Unfortunately, the records from our animal facility showed that these mice used in our studies in 2012–2013 were exposed to mouse *norovirus*, *H.* sp., and *H. hepaticus*. For a comparison, we analyzed male iPLA2β-null mice at 12 months of age which were housed under pathogen-free conditions (22). These mutants did not show any changes in body weights and % liver and subcutaneous fat (**Figure 1A**), liver histology (**Figure 1B**), and hepatic TG and NEFA levels (**Figure 1C**). However, pathogen-exposed male mutant mice at 9–14 months old displayed significant reduction in body weights and % liver and subcutaneous fat (**Figure 1D**), the numbers of lipid droplets seen in liver histology (**Figure 1E**), and hepatic TG and Chol levels (**Figure 1G**). Noticeably, pathogen-exposed WT mice exposed to natural pathogens showed the presence of lipid droplets (**Figure 1E**) which were not seen under pathogen-free conditions (**Figure 1B**). The reduction of % subcutaneous fat in pathogen-exposed mutants (**Figure 1D**) was caused by a decrease in TG, NEFA, and GPL (**Figure 1F**). Similar to male mutants under pathogen-free conditions (22), the mutants exposed to pathogens did not show any changes in the total hepatic GPL subclasses (PC, PE, PS, and PI) (**Figure 1G**). Since *PLA2G6* gene is regulated by sterol regulatory element-binding protein1 (*SREBP1*) (31), we therefore analyzed genes related to *de novo* lipogenesis in our pathogen-exposed cohort. Mutant mice displayed significant downregulation of hepatic fatty acid synthase (Fasn) and elongation of very long-chain fatty acid protein6 (Elovl6) protein (**Figure 1H**) as well as *Srebp1c* and *Fasn* mRNA (**Figure 1I**). Interestingly, mRNA expression of TG synthesis gene diacylglycerol acyltransferase1 (*Dgat1*) and lipolysis gene adipose triglyceride lipase (*Atgl*) was upregulated in mutant livers. This upregulation suggested a possible feedback response against downregulation of *de novo* lipogenesis and suppression of hepatic TG (**Figure 1G**).

To link metabolic changes with hepatocellular injury, we performed investigation in hepatocytes isolated from pathogen-exposed male WT and iPLA2β-null mice at 2 months of age. Consistent with liver data (**Figure 1I**), hepatocytes from iPLA2β-null mice displayed downregulation of *Srebp1c*, *Fasn*, and acetyl-CoA carboxylase (*Acc1*) (**Figure 1J**). This downregulation was concomitant with upregulated expression of chemokine monocyte chemoattractant protein-1 (*Mcp-1*) and fibrosis gene plasminogen activator inhibitor-1 (*Pai-1*). Upon treatment with palmitate (Pal) to induce lipotoxicity, mutant hepatocytes showed a significant decrease in cellular Chol, but without any changes in TG (**Figure 1K**). Pal treatment of WT hepatocytes led to upregulation of apoptosis cleaved poly(ADP-ribose) polymerase-1 (Parp-1), p53 up-regulated modulator of apoptosis (Puma), and C/EBP homologous protein (Chop) protein (**Figure 1L**), which were further upregulated in Pal-treated hepatocytes from mutant mice. Without Pal treatment, mutant hepatocytes already showed increased expression of cleaved Parp-1 (**Figure 1L**). Thus, even though *de novo* lipogenesis was suppressed, Pal-induced apoptosis in mutant hepatocytes may occur independent of cellular TG levels.

### 3.2 Suppressed hepatic lipid synthesis in male iPLA2β-null mice exposed to natural pathogens

**Figure 2:**
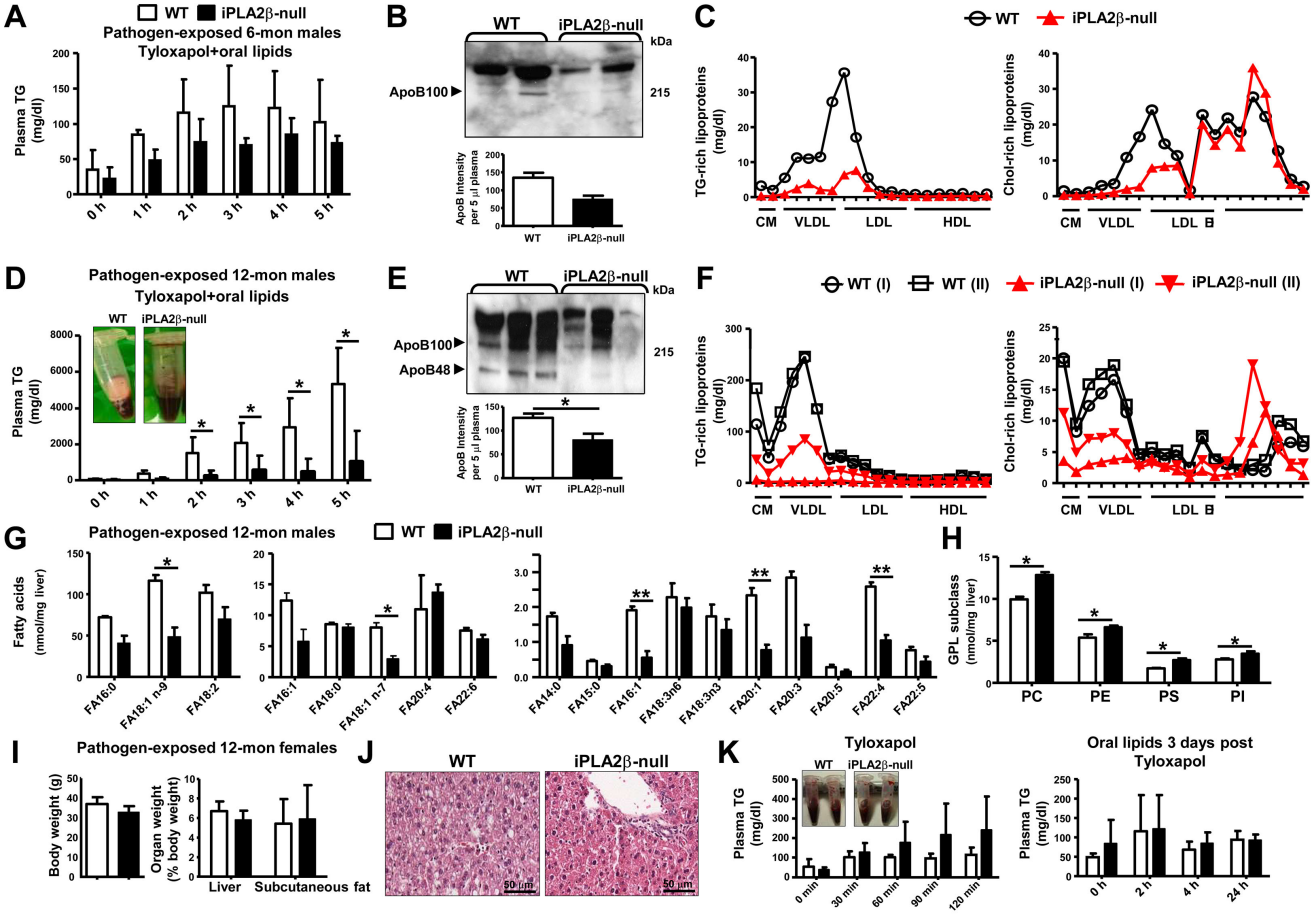
Male iPLA2β-null mice exposed to pathogens displayed suppressed hepatocellular lipid synthesis and lipoprotein secretion associated with suppressed FA but accumulated GPL. Pathogen-exposed male and female WT and iPLA2β-null mice at 6 or 12 months of age were used in this study. **(A)** Hepatic synthesis using tyloxapol and oral lipids was investigated in male mice at 6 months old. Plasma TG levels prior to and every hour after lipid treatment for 5 h (N = 4). At 48 h post tyloxapol and lipid treatment, **(B)** Western blot analysis of plasma ApoB100; and **(C)** the profiles of plasma TG- and Chol-rich lipoproteins (CM, VLDL, LDL, HDL) in a pooled plasma sample from 4 mice per genotype. **(D)** Hepatic synthesis was investigated in male mice at 12 months old. Plasma TG levels prior to and every hour after lipid treatment for 5 h (N = 4). At 5 h post tyloxapol and lipid treatment, **(E)** Western blot analysis of plasma ApoB48 and ApoB100; **(F)** the profiles of plasma TG- and Chol-rich lipoproteins in two pooled plasma samples (indicated as I and II) from 2 mice per genotype; **(G)** the profiles of hepatic individual FA as determined by GC/MS (N = 3); and **(H)** the profiles of hepatic PC, PE, PS, and PI as determined by ESI/MS-MS (N = 3). For pathogen-exposed female mice at 12 months of age, **(I)** body weights (g) and the percentage of liver and subcutaneous fat per body weights (N = 4-6); **(J)** representative H&E staining of livers, and **(K)** plasma TG levels at 30 min, 60 min, 90 min, and 120 min post tyloxapol treatment (left panel) as well as at 2 h, 4 h, and 24 h after oral administration of lipids performed 3 days post tyloxapol treatment (right panel) (N = 5-6). Data are mean ± SD. *p < 0.05 and **p < 0.01 by two-tailed Mann Whitney U tests.

In an absence of external sources of FA, it is shown that iPLA2β provides FA for TG synthesis in cultured cells (24). We therefore investigated hepatic TG synthesis in our pathogen-exposed cohort which was assessed by treatment with a lipoprotein-lipase inhibitor tyloxapol with or without oral administration of lipids, and TG levels were measured in blood from tail vein (26). We first utilized male WT and iPLA2β-null mice at 6 months of age. At 1–5 h post tyloxapol+oral lipid treatment, these mutants showed a trend toward reduction of plasma TG levels (**Figure 2A**). At 48 h post lipid treatment, these mutants however showed a reduction in plasma ApoB100, the major lipoprotein released by liver (**Figure 2B**) as well as plasma TG- and Chol-rich VLDL/LDL (**Figure 2C**). For the next cohort, we utilized mice at 12 months of age. Remarkably, these mutants showed a significant reduction of plasma TG levels at 2–5 h post tyloxapol+lipid treatment (**Figure 2D**). At 5 h post lipid treatment, protein expression of plasma ApoB100 (released by liver) and ApoB48 (released by intestine) was also decreased in these mutants (**Figure 2E**). In line with ApoB data, mutants displayed the reduction of TG- and Chol-rich CM, VLDL, and LDL levels (**Figure 2F**). Upon mass spectrometric profiling of liver samples taken at 5 h post lipid treatment, these mutants showed attenuated hepatic levels of many FA species (**Figure 2G**). We previously showed that hepatic levels of individual GPL species containing polyunsaturated fatty acids were increased in pathogen-free male mutants (21, 22). Under lipid-overload conditions induced by tyloxapol+oral lipids, the increase was more obvious in pathogen-exposed mutants showing a significant increase of all GPL subclasses PC, PE, PS, and PI (**Figure 2H**). Moreover, the suppressive response on FA and TG synthesis by iPLA2β inactivation was greater with age (**Figure 2A** versus **2D**) correlating with liver fibrosis seen in aged male iPLA2β-null mice housed in pathogen-free conditions (18).

Because female mice were used to expand colonies during 2011-2013, we were able to perform limited metabolic experiments from these mice. Unlike male counterparts (**Figure 1D**), pathogen-exposed female mutants at 12 months of age did not show any changes in body weight and % liver and subcutaneous fat (**Figure 2I**). Compared to male counterparts (**Figure 1D**), these female mutants showed lesser numbers and smaller sizes of lipid droplets together with significant infiltration of immune cells seen at the portal tracts (**Figure 2J**). Within the first 120 min after tyloxapol treatment, mutant mice showed only a trend increase in plasma TG levels when compared to WT (**Figure 2K**). Upon oral administration of lipids performed 3 days post tyloxapol treatment, these same mice showed no difference in plasma TG levels. Hence, global *PLA2G6* deficiency combined with pathogen exposure attenuated body and liver weights together with suppressed hepatic lipid synthesis seen in male but not female mice.

### 3.3 Natural pathogen-exposed iPLA2β-null mice of both sexes displayed hepatocellular inflammatory fibrosis

**Figure 3:**
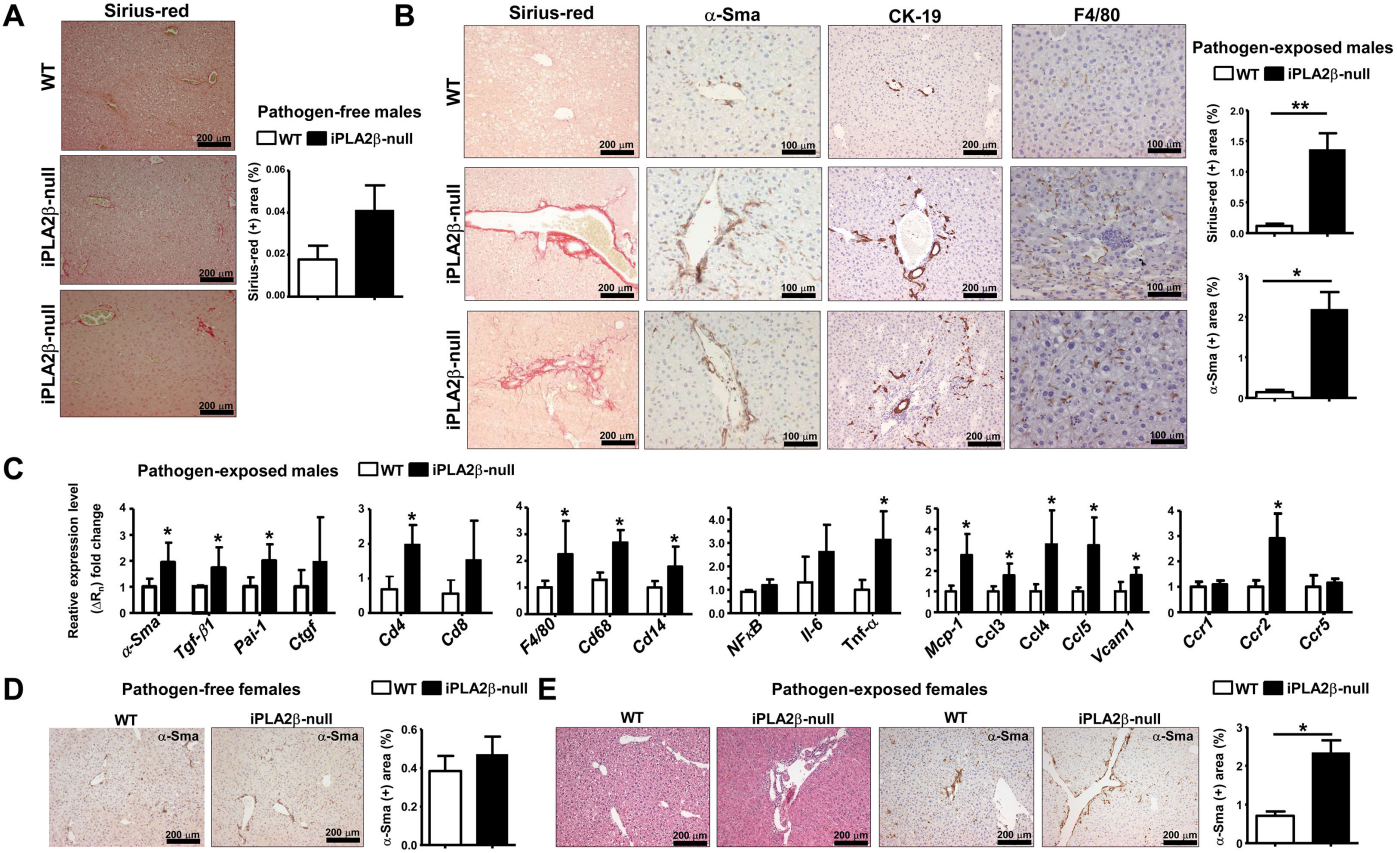
Pathogen-exposed iPLA2β-null mice of both sexes displayed hepatocellular inflammatory fibrosis. Inflammatory fibrosis was analyzed in pathogen-free and naturally infected male and female WT and iPLA2β-null mice at 9–14 months of age. **(A)** Representative images (left) and quantification (right) of hepatic Sirius-red staining from pathogen-free male mice. For pathogen-exposed male mice, **(B)** representative images of hepatic Sirius-red staining (quantification, N = 4-7), α-Sma (quantification, N = 4-5), CK-19, and F4/80 IHC-staining; and **(C)** RT-qPCR analysis of hepatic gene expression of fibrogenesis *α-Sma*, *Tgf-β1*, *Pai-1*, and *Ctgf*; lymphocyte markers *Cd4* and *Cd8*; granulocyte markers *F4/80*, *Cd68*, and *Cd14*; cytokines *NF-kB*, *Il-6*, *Tnf-α; chemokines Mcp-1*, *Ccl3*, *Ccl4*, *Ccl5*, and *Vcam-1*; and chemokine receptors *Ccr1*, *Ccr2*, and *Ccr5* (N = 4-7). **(D)** Hepatic α-Sma staining (quantification, N = 5-7) in pathogen-free female mice. **(E)** Hepatic H&E and α-Sma staining (quantification, N = 4-5) in pathogen-exposed female mice. Data are mean ± SD. *p < 0.05 and **p < 0.01 by two-tailed Mann Whitney U tests.

We further performed hepatic fibrosis analysis of pathogen-exposed male and female mice at 9–14 and 12 months old, respectively. Male iPLA2β-null mice housed under pathogen-free conditions (22) did not show any significant changes in hepatic fibrosis as determined by Sirius-Red staining (**Figure 3A**). However, pathogen-exposed male mutants displayed a significant increase in positive staining of hepatic Sirius-Red, α-smooth muscle actin (α-Sma), cytokeratin-19 (CK-19), and F4/80 (**Figure 3B**). These mutants also showed upregulation of fibrosis genes *(α-Sma*, *Tgf-β1*, and *Pai-1*), immune cell markers (*Cd4*, *F4/80*, *Cd68*, and *Cd14*), and cytokines/chemokines/chemokine receptors (*Tnf-α, Mcp-1*, *Ccl3*, *Ccl4*, *Ccl5*, *Vcam1*, and *Ccr2*) (**Figure 3C**). Thus, the suppression of hepatic TG and plasma lipoproteins (**Figures 1**, **2**) in male iPLA2β-null mice was associated with exaggerated hepatic inflammatory fibrosis during natural infection.

Upon analyzing chow-fed female mice housed under pathogen-free conditions in our 2019 publication (32), iPLA2β-null mice did not show any significant changes in hepatic fibrosis as determined by α-Sma staining (**Figure 3D**). Together with hepatic immune cell infiltration (**Figure 2J**), pathogen-exposed female mutants displayed significant increase in hepatic fibrosis as observed in H&E and α-Sma staining (**Figure 3E**). Thus, female mutants under natural pathogen exposure exhibited inflammatory fibrosis which was independent of hepatic lipid synthesis (**Figure 2K**).

### 3.4 Susceptibility of pathogen-exposed iPLA2β-null mice of both sexes towards liver injury induced by low-dose endotoxin LPS

**Figure 4:**
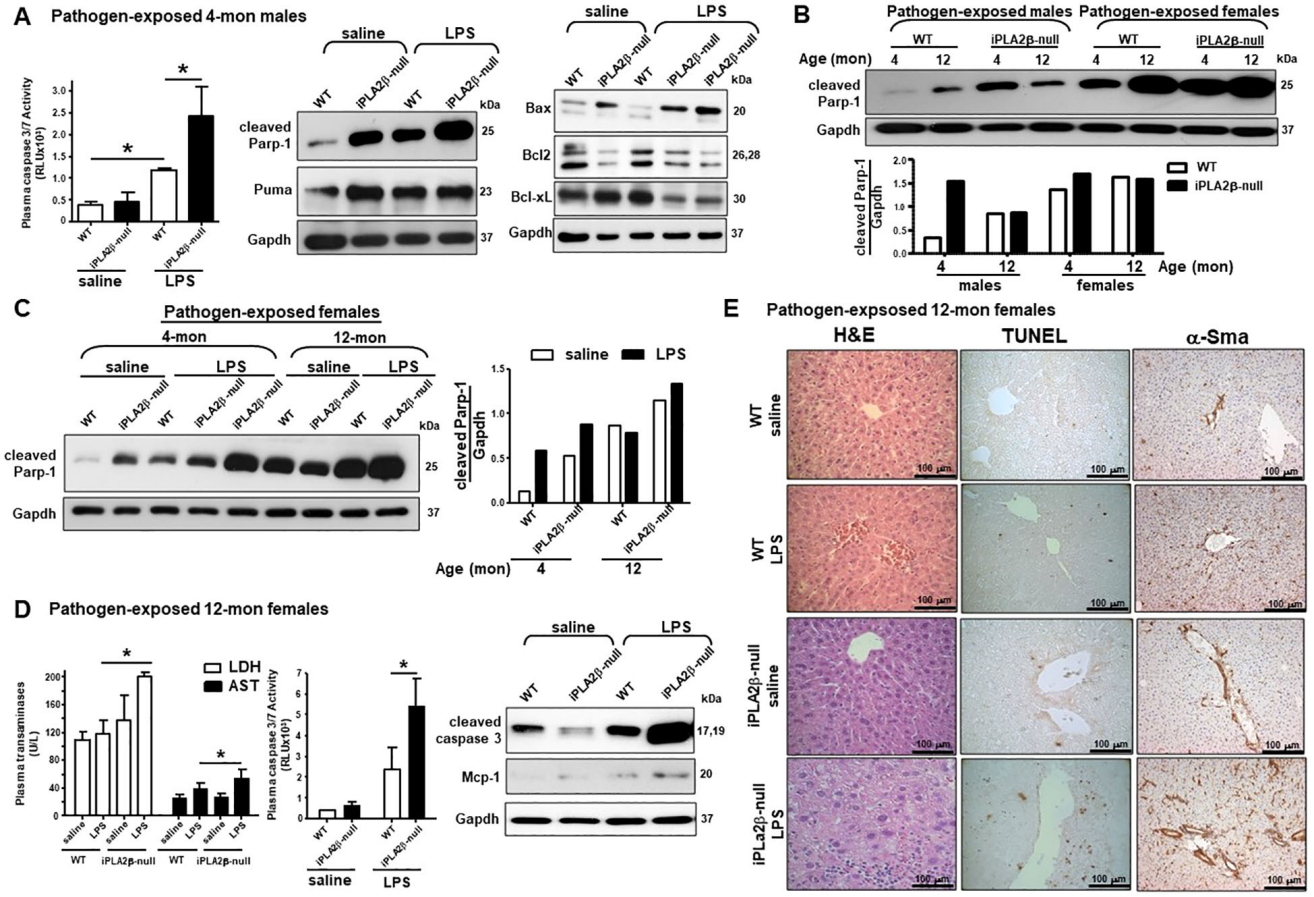
Susceptibility of pathogen-exposed iPLA2β-null mice of both sexes towards liver injury induced by endotoxin. Fasted pathogen-exposed male and female WT and iPLA2β-null mice at 4 and 12 months of age were intraperitoneally injected with saline or 1 mg/kg LPS for 16 h. **(A)** Left panel shows plasma caspase 3 activity from 4-month-old male mice treated with saline or LPS (N = 4). Right panel shows representative hepatic protein expression of cleaved Parp-1, Puma, Bax, Bcl2, and Bcl-xL. **(B)** Hepatic expression (top) and quantification (bottom) of cleaved Parp-1 from male and female mice at 4 and 12 months of age. **(C)** Hepatic expression (left) and quantification (right) of cleaved Parp-1 from 4- and 12-month-old female mice treated with saline or LPS. **(D)** Left panel shows plasma LDH (white bars) and AST (black bars) levels from 12-month-old female mice treated with saline or LPS (N = 4). Middle panel shows plasma caspase 3 activity. Right panel shows representative hepatic protein expression of cleaved caspase 3 and Mcp-1. **(E)** Representative H&E staining, TUNEL, and α-Sma IHC staining in livers of 12-month-old female mice treated with saline or LPS. Data were presented as mean ± SD, *p < 0.05 by two-tailed Mann Whitney U.

Upon the completion of studies using male mice (**Figures 1**-**3**), female mice were relieved from breeding rounds, and we were able to obtain small cohorts of male and female mice at 4 and 12 months of age for further studies. Under the background of natural pathogen exposure, we aimed to determine whether iPLA2β-null mice would be susceptible to an additional injury induced by endotoxin LPS. We performed an intraperitoneal injection of mice with 1 mg/kg *E. Coli* LPS for 16 h. This LPS dosage induces mild systemic inflammation, and the 16 h time-point represents an acute innate immune response to LPS. In 4-month-old pathogen-exposed male mice, LPS treatment of WT led to an increase in apoptosis as seen in plasma caspase 3/7 activity and hepatic protein expression of cleaved Parp-1, Puma, and Bax (**Figure 4A**). These apoptosis markers were further upregulated in iPLA2β-null mice while expression of anti-apoptosis Bcl2 and Bcl-xL was concomitantly downregulated. Noticeably, saline-treated mutants already displayed upregulation of cleaved Parp-1, Puma, and Bax concomitant downregulation of Bcl2, indicating that natural-pathogen infection may lead to hepatocellular injury in male mutant mice under basal conditions.

Because of stark LPS susceptibility on apoptosis in 4-month-old male mutants (**Figure 4A**), we compared the extent of hepatic apoptosis among male and female mice at 4 and 12 months of age. We then compared LPS susceptibility among female mice at 4 and 12 months of age. Compared to WT, iPLA2β-null mice of both sexes at 4 months of age displayed stark upregulated expression of apoptosis cleaved Parp-1 (**Figure 4B**). Mutants of both sexes at 4 months of age showed greater apoptosis response than those at 12 months of age. For both genotypes, female mice at 4 and 12 months old showed increased cleaved Parp-1 expression, compared to age-matched males. Upon evaluation of apoptosis susceptibility induced by LPS, female mice of both genotypes at 4 months of age were similarly susceptible to LPS-induced apoptosis (**Figure 4C**). However, female mutants at 12 months of age were more susceptible to LPS-induced apoptosis than age-matched WT (**Figure 4C**). Hence, adult mutants of both sexes exhibited increased apoptotic injury which was exacerbated by LPS treatment.

To confirm LPS susceptibility on apoptosis in female mutants at 12 months of age (**Figure 4C**), we further characterized liver injury in greater details. Compared to LPS-treated WT, LPS-treated mutants displayed an elevation of plasma LDH, AST, and caspase 3/7 activity concomitant with an upregulation of cleaved caspase 3 and Mcp-1 protein (**Figure 4D**). Consistent with immune cell infiltration (**Figure 2J**) as well as hepatic fibrosis and α-Sma staining (**Figure 3E**), female mutants under saline treatment already displayed wider sinusoid space and larger size of hepatocyte nuclei (**Figure 4E**). After LPS treatment, mild liver injury with red blood cell accumulation was observed in WT mice, while mutant mice showed marked hepatic infiltration of immune cells, particularly, phagocytes (bottom panel, **Figure 4E**). The exaggeration of apoptosis and fibrosis was consistently observed in livers of LPS-treated mutants as demonstrated by increased staining of TUNEL and α-Sma, respectively. Taken together, male and female iPLA2β-null mice at 4 months of age displayed increased hepatic apoptosis and LPS susceptibility when compared to WT counterparts. Compared to WT counterparts, female mutants at 12 months old exhibited hepatic inflammatory fibrosis induced by LPS. Thus, global *PLA2G6* deficiency under pathogen exposure rendered susceptibility to LPS-induced injury in adult mice of both sexes.

## 4 Discussion

We here demonstrated physiological role of *PLA2G6* in maintaining hepatic homeostasis in mice which were exposed to mouse *norovirus*, *Helicobacter* sp., and *Helicobacter hepaticus*. As expected, the reported phenotypes of adult iPLA2β-null mice at 9–14 months of age were greater than those reported in mutants which were housed in clean mouse rooms (22, 32). Under pathogen exposed conditions, male mutants displayed defective hepatic glycerolipid metabolism, reduced hepatic TG, induction of hepatocellular apoptosis and fibrosis, and susceptibility to LPS injury. Despite no changes in hepatic lipid synthesis, female mutants also displayed hepatic fibrosis and susceptibility to LPS injury. Thus, global *PLA2G6* deficiency under pathogen exposure resulted in alteration in hepatic glycerolipid metabolism in a male-biased manner, while inflammatory hepatic fibrosis and susceptibility to LPS injury was exacerbated in both sexes.

Global *PLA2G6* inactivation leads to defective global lipidomic remodeling in all cell types. *PLA2G6* deficiency in adipocytes may lead to suppression of adipocyte differentiation (33), and this could result in decreased subcutaneous weights and lipids seen in male iPLA2β-null mice. Suppressed adipocyte biogenesis may result in impaired secretion of anti-fibrotic adipokines such as adiponectin (34), and such suppression may allow increased liver fibrosis seen in male mutants. *PLA2G6* deficiency in hepatocytes may lead to the reduction of lysoPC which is a ‘find-me’ signal for the removal of dying hepatocytes (35). In livers of male (21, 22) and female (32) mutants, this deficiency leads to an increase of individual GPL containing polyunsaturated fatty acids which could be subjected to peroxidation and consequently resulting in hepatic inflammation (36). Moreover, *PLA2G6* deficiency in macrophages may impair chemotaxis towards dying hepatocytes (37). Thus, PLA2G6 deficiency in hepatocytes and macrophages would likely result in accumulation of dying hepatocytes that could lead to hepatic inflammation and consequently fibrotic injury observed in iPLA2β-null mice in our study. Supporting this, activation of Kupffer cells from male iPLA2β-null mice with elevated release of IL-6 was observed during anti-CD95 antibody-induced liver injury (20). In line with this, *PLA2G6* deficiency specifically in myeloid cells in male mice resulted in an increase in plasma IL-6 and susceptible to LPS injury (38). Notably, these myeloid-specific mutants of both sexes displayed increased hepatic necrosis and accumulation of mononuclear cells at 27% and 57% rates for males and females, respectively (38). These abnormalities were associated with an increase in individual GPL in mutant bone-marrow-derived macrophages, thus correlating altered GPL metabolism with immune activation. While C57BL/6 mice are typically resistant to damage caused by *H. hepaticus* (39), we surmise that PLA2G6 deficiency in myeloid cells may likely contribute to the susceptibility to active hepatic fibrosis in iPLA2β-null mice of both sexes infected with this pathogen. This also highlights the detrimental role of PLA2G6-deficient myeloid cells and macrophages as a common contributor to tissue injury as reported in aged male mutants under pathogen-free conditions (18) as well as pathogen-exposed female (19) and male (20) mutants undergoing acute liver injury.

In line with previous report in cultured cells (24), we provided novel findings that male iPLA2β-null mice under pathogen exposure showed suppression of hepatic TG and Chol under basal conditions as well as suppression of hepatic lipid synthesis under lipid overload induced by tyloxapol+oral lipids. This suppression was associated downregulation of *de novo* lipogenic genes Fasn/Elovl6, *Srebp1c*, and *Fasn* in liver and hepatocytes of mutant mice. Under pathogen-free conditions, male iPLA2β-null mice however did not show any alterations in hepatic TG and expression of Fasn and Elovl6 protein (22). This indicates that the suppression of *de novo* lipogenesis in male mutants was likely due to infection with rodent pathogens. We surmise that, via inter-organ communication, activated macrophages from iPLA2β-null mice during stress induced by pathogens may inhibit hepatic *de novo* lipogenesis pathways (40). As for pathogen-exposed male mutants, the suppression of hepatic TG (41) and plasma lipoproteins (42) may also render the susceptibility of these mice to hepatic apoptosis and LPS-induced liver injury, respectively. Moreover, we observed that pathogen-exposed male mutants showed a decrease in Chol levels in the liver, hepatocytes, and plasma Chol-rich lipoproteins. As hepatic FASN regulates Chol metabolism (43), thus downregulation of Fasn in these mutants may result in reduction of Chol synthesis. In addition, the changes in proportions of PE and PC in mutant hepatocytes may alter expression of the scavenger receptor class B type I, an enzyme that plays a role in cholesterol uptake and secretion at the hepatocyte basolateral membrane (44). Noticeably, mutant hepatocytes with or without Pal treatment did not alter hepatocellular TG levels (**Figure 1K**). Plausibly, an increase of some individual GPL in mutants (21, 22) may in turn be a source of hepatocellular TG (3) thus counteracting the expected TG decrease. As TG levels in mutant hepatocytes were not correlated with apoptosis, it is possible that PLA2G6 deficiency itself may increase susceptibility to palmitate-induced ER stress/apoptosis independently of cellular TG levels. Finally, the suppression of TG- and Chol-rich lipoproteins and concomitant hepatic fibrosis in male mutants was in line with low levels of circulating lipoproteins reported in patients with primary biliary cirrhosis (45) and cirrhosis (46). Thus, the impairment of hepatic lipid synthesis and lipoprotein secretion by global *PLA2G6* inactivation in male mice may be indicators for underlying chronic liver disease.

Unlike male counterparts, pathogen-exposed female iPLA2β-null mice showed no alterations in body weights, % liver weights, and hepatic TG synthesis under tyloxapol treatment. While the expression of *Srebp1c* and *Fasn* was not altered in pathogen-free female mutants (32), unfortunately, we did not have data on *de novo* lipogenesis genes in female mutants under pathogen exposure. The mechanism for lack of hepatic TG synthesis in female mutants may rely on the effects of female hormones on hepatic *de novo* lipogenesis. Relevantly, it is shown that progesterone increases *de novo* lipogenic gene expression in adipose tissues (47), liver, and plasma lipids (48) in female mice. Such increase may counteract the expected reduction of adipose/liver weights and suppression of metabolic parameters resulting in no overall changes seen in pathogen-exposed female mutants. Progesterone is a steroid hormone secreted by the granulosa luteal cells of the ovary. We previously reported that bone-marrow-derived macrophages from myeloid-specific PLA2G6-deficient female mice displayed elevated levels of AA (49). As AA reportedly induces progesterone release from luteum (50), speculatively, *PLA2G6* deficiency in endometrial epithelial cells may release elevated levels of AA which can in turn induce progesterone release to blood and consequently resulting in increased hepatic *de novo* lipogenesis (48). Progesterone-related pathways may represent one possible mechanism underlying the observed sex-dependent differences in the alterations of metabolic parameters, and that further experiment measuring circulating or hepatic progesterone levels would be required to test this possibility.

While not seen under pathogen-free conditions, mutants of both sexes at 12 months of age exhibited hepatic fibrosis during pathogen exposure (**Figure 3**). Of note, pathogen-exposed mutants of both sexes at 4 months of age showed a greater apoptosis response of cleaved Parp-1 than those at 12 months of age (**Figures 4B, C**), which is contrast to the increased hepatic fibrosis in aged mutants under pathogen-free conditions (18). This result suggests that prolonged exposure of pathogens in mutants for 12 months may elicit apoptosis resistance compared to those exposed to pathogens for 4 months. As our mice were exposed to *norovirus*, a possibility could be that, the murine *norovirus* protein, so-called open reading frame4, is reportedly able to antagonize the innate immune response to infection by delaying the upregulation of cellular genes activated by the innate pathway (51). In addition, after prolonged exposure to *norovirus*, the adaptive response and lymphopoiesis induced by macrophages from mutants (38) may become dominant, which can result in apoptosis resistance. The mechanisms underlying the age-dependent difference remain unresolved, and they may involve age-dependent adaptation, altered immune responses after prolonged pathogen exposure, or cohort-specific effects. Additional experiments are required to determine these mechanisms. Furthermore, we observed that female mice of both genotypes at 4 and 12 months old showed increased cleaved Parp-1 expression, compared to age-matched males (**Figure 4B**). This result is in line with the reported detrimental effects of progesterone in activating fibrogenesis pathways (52) and exacerbating drug-induced liver injury (53). Thus, progesterone appears to be involved in metabolic (47, 48) and inflammatory (52, 53) dysfunctions in female mutants. Finally, the worsened apoptosis in female mutants with or without LPS treatment, compared to WT counterparts (**Figure 4C**) could likely be due to the inability of PLA26-deficient hepatocytes and macrophages (35–37) to remove dying hepatocytes as discussed above.

*PLA2G6* is upregulated in human brain during normal aging, while its expression is downregulated in Alzheimer’s disease (54). Indeed, the mutations in PLA2G6 gene lead to neurodegenerative disorders (12, 13). Similar to the brain, hepatocellular *PLA2G6* may likely be protective over mouse lifetime. Global *PLA2G6* deficiency in iPLA2β-null mice led to abnormalities in the brain (14) and the liver under pathogen exposure as presented in our study. Our findings also suggest a potential link between neurodegeneration and liver dysfunction, possibly mediated by shared metabolic and inflammatory pathways involving *PLA2G6* inactivation. Interestingly, patients with dementia often exhibit liver dysfunction (55) and hepatic cirrhosis (56). On the other hand, we previously reported that iPLA2β-null mice were protected against genetic (21) and high-fat diet (22)-induced obesity and fatty liver by replenishing the loss of GPL. This indicates that *PLA2G6* mediates hepatic lipid metabolism and inflammation depending on biological and disease context. With this notion, *PLA2G6* may be an important target for drug development with a consideration of cell-specific lipid metabolism and disease context (57).

In conclusion, our work provides novel insights into the pivotal role of *PLA2G6* in hepatocellular glycerolipid synthesis and metabolism in male mice under pathogen exposed conditions, and that global inactivation of this gene increased hepatic fibrosis and susceptibility to LPS-induced injury in both sexes. This gene can be used as a target to assist in the sex-dimorphic development of therapeutic strategies in promoting healthy aging and treatment of infection-induced hepatic fibrosis.

## Data Availability

Analyzed data on lipidomic profiling (fatty acids and phospholipids) can be found at https://doi.org/10.6084/m9.figshare.31834393. Other data will be available upon request to WC: Walee.Chamulitrat@med.uni-heidelberg.de.

## References

[1] Moustafa T Fickert P Magnes C Guelly C Thueringer A Frank S . Alterations in lipid metabolism mediate inflammation, fibrosis, and proliferation in a mouse model of chronic cholestatic liver injury. Gastroenterology. (2012) 142:140–151.e12. doi: 10.1053/j.gastro.2011.09.051 22001865

[2] Li Z Agellon LB Allen TM Umeda M Jewell L Mason A . The ratio of phosphatidylcholine to phosphatidylethanolamine influences membrane integrity and steatohepatitis. Cell Metab. (2006) 3:321–31. doi: 10.1016/j.cmet.2006.03.007 16679290

[3] van der Veen JN Lingrell S Vance DE . The membrane lipid phosphatidylcholine is an unexpected source of triacylglycerol in the liver. J Biol Chem. (2012) 287:23418–26. doi: 10.1074/jbc.M112.381723 22610093 PMC3390618

[4] Jacobs RL Lingrell S Zhao Y Francis GA Vance DE . Hepatic CTP:phosphocholine cytidylyltransferase-alpha is a critical predictor of plasma high density lipoprotein and very low density lipoprotein. J Biol Chem. (2008) 283:2147–55. doi: 10.1074/jbc.M706628200 18042552

[5] Six DA Dennis EA . The expanding superfamily of phospholipase A(2) enzymes: classification and characterization. Biochim Biophys Acta. (2000) 1488:1–19. doi: 10.1016/S1388-1981(00)00105-0 11080672

[6] Turk J White TD Nelson AJ Lei X Ramanadham S . iPLA2β and its role in male fertility, neurological disorders, metabolic disorders, and inflammation. Biochim Biophys Acta Mol Cell Biol Lipids. (2019) 1864:846–60. doi: 10.1016/j.bbalip.2018.10.010 30408523 PMC6432790

[7] Lu Y Day FR Gustafsson S Buchkovich ML Na J Bataille V . New loci for body fat percentage reveal link between adiposity and cardiometabolic disease risk. Nat Commun. (2016) 7:10495. doi: 10.1038/ncomms10495 26833246 PMC4740398

[8] Huang LO Loos RJF Kilpeläinen TO . Evidence of genetic predisposition for metabolically healthy obesity and metabolically obese normal weight. Physiol Genomics. (2018) 50:169–78. doi: 10.1152/physiolgenomics.00044.2017 29341865 PMC6048453

[9] Teslovich TM Musunuru K Smith AV Edmondson AC Stylianou IM Koseki M . Biological, clinical and population relevance of 95 loci for blood lipids. Nature. (2010) 466:707–13. doi: 10.1038/nature09270 20686565 PMC3039276

[10] Kocarnik JM Richard M Graff M Haessler J Bien S Carlson C . Discovery, fine-mapping, and conditional analyses of genetic variants associated with C-reactive protein in multiethnic populations using the Metabochip in the Population Architecture using Genomics and Epidemiology (PAGE) study. Hum Mol Genet. (2018) 27:2940–53. doi: 10.1093/hmg/ddy211 29878111 PMC6077792

[11] Tremblay BL Rudkowska I Couture P Lemieux S Julien P Vohl MC . Modulation of C-reactive protein and plasma omega-6 fatty acid levels by phospholipase A2 gene polymorphisms following a 6-week supplementation with fish oil. Prostaglandins Leukot Essent Fatty Acids. (2015) 102–103:37–45. doi: 10.1016/j.plefa.2015.10.002 26525102

[12] Guo YP Tang BS Guo JF . PLA2G6-associated neurodegeneration (PLAN): review of clinical phenotypes and genotypes. Front Neurol. (2018) 9:1100. doi: 10.3389/fneur.2018.01100 30619057 PMC6305538

[13] Kurtovic-Kozaric A Singer-Berk M Wood J Evangelista E Panwala L Hope A . An estimation of global genetic prevalence of PLA2G6-associated neurodegeneration. Orphanet J Rare Dis. (2024) 19:388. doi: 10.1186/s13023-024-03275-x 39425167 PMC11489993

[14] Blanchard H Taha AY Cheon Y Kim HW Turk J Rapoport SI . iPLA2β knockout mouse, a genetic model for progressive human motor disorders, develops age-related neuropathology. Neurochem Res. (2014) 39:1522–32. doi: 10.1007/s11064-014-1342-y 24919816 PMC4364003

[15] Bao S Miller DJ Ma Z Wohltmann M Eng G Ramanadham S . Male mice that do not express group VIA phospholipase A2 produce spermatozoa with impaired motility and have greatly reduced fertility. J Biol Chem. (2004) 279:38194–200. doi: 10.1074/jbc.M406489200 15252026 PMC3733543

[16] Bao S Song H Wohltmann M Ramanadham S Jin W Bohrer A . Insulin secretory responses and phospholipid composition of pancreatic islets from mice that do not express Group VIA phospholipase A2 and effects of metabolic stress on glucose homeostasis. J Biol Chem. (2006) 281:20958–73. doi: 10.1074/jbc.M600075200 16732058 PMC2044498

[17] Ramanadham S Yarasheski KE Silva MJ Wohltmann M Novack DV Christiansen B . Age-related changes in bone morphology are accelerated in group VIA phospholipase A2 (iPLA2beta)-null mice. Am J Pathol. (2008) 172:868–81. doi: 10.2353/ajpath.2008.070756 18349124 PMC2276416

[18] Jiao L Gan-Schreier H Zhu X Wei W Tuma-Kellner S Liebisch G . Ageing sensitized by iPLA2β deficiency induces liver fibrosis and intestinal atrophy involving suppression of homeostatic genes and alteration of intestinal lipids and bile acids. Biochim Biophys Acta Mol Cell Biol Lipids. (2017) 1862:1520–33. doi: 10.1016/j.bbalip.2017.09.001 28888832

[19] Jiao L Gan-Schreier H Tuma-Kellner S Stremmel W Chamulitrat W . Sensitization to autoimmune hepatitis in group VIA calcium-independent phospholipase A2-null mice led to duodenal villous atrophy with apoptosis, goblet cell hyperplasia and leaked bile acids. Biochim Biophys Acta. (2015) 1852:1646–57. doi: 10.1016/j.bbadis.2015.04.025 25957555

[20] Inhoffen J Tuma-Kellner S Straub B Stremmel W Chamulitrat W . Deficiency of iPLA_2_β primes immune cells for proinflammation: potential involvement in age-related mesenteric lymph node lymphoma. Cancers (Basel). (2015) 7:2427–42. doi: 10.3390/cancers7040901 26690222 PMC4695901

[21] Deng X Wang J Jiao L Utaipan T Tuma-Kellner S Schmitz G . iPLA2β deficiency attenuates obesity and hepatic steatosis in ob/ob mice through hepatic fatty-acyl phospholipid remodeling. Biochim Biophys Acta. (2016) 1861:449–61. doi: 10.1016/j.bbalip.2016.02.004 26873633

[22] Otto AC Gan-Schreier H Zhu X Tuma-Kellner S Staffer S Ganzha A . Group VIA phospholipase A2 deficiency in mice chronically fed with high-fat-diet attenuates hepatic steatosis by correcting a defect of phospholipid remodeling. Biochim Biophys Acta Mol Cell Biol Lipids. (2019) 1864:662–76. doi: 10.1016/j.bbalip.2019.01.012 30735855

[23] Hayashi D Dennis EA . Molecular basis of unique specificity and regulation of group VIA calcium-independent phospholipase A2 (PNPLA9) and its role in neurodegenerative diseases. Pharmacol Ther. (2023) 245:108395. doi: 10.1016/j.pharmthera.2023.108395 36990122 PMC10174669

[24] Gubern A Barceló-Torns M Casas J Barneda D Masgrau R Picatoste F . Lipid droplet biogenesis induced by stress involves triacylglycerol synthesis that depends on group VIA phospholipase A2. J Biol Chem. (2009) 284:5697–708. doi: 10.1074/jbc.M806173200 19117952

[25] Tran K Wang Y DeLong CJ Cui Z Yao Z . The assembly of very low density lipoproteins in rat hepatoma McA-RH7777 cells is inhibited by phospholipase A2 antagonists. J Biol Chem. (2000) 275:25023–30. doi: 10.1074/jbc.M908971199 10827200

[26] Millar JS Cromley DA McCoy MG Rader DJ Billheimer JT . Determining hepatic triglyceride production in mice: comparison of poloxamer 407 with Triton WR-1339. J Lipid Res. (2005) 46:2023–8. doi: 10.1194/jlr.D500019-JLR200 15995182

[27] Toshima G Iwama Y Kimura F Matsumoto F Miu F Takahashi J . LipoSEARCH®; analytical GP-HPLC method for lipoprotein profiling and its applications. J Biol Macromol. (2013) 13:21–32. doi: 10.14533/jbm.13.21

[28] Ecker J Scherer M Schmitz G Liebisch G . A rapid GC-MS method for quantification of positional and geometric isomers of fatty acid methyl esters. J Chromatogr B Analyt Technol BioMed Life Sci. (2012) 897:98–104. doi: 10.1016/j.jchromb.2012.04.015 22542399

[29] Liebisch G Scherer M . Quantification of bioactive sphingo- and glycerophospholipid species by electrospray ionization tandem mass spectrometry in blood. J Chromatogr B Analyt Technol BioMed Life Sci. (2012) 883–884:141–6. doi: 10.1016/j.jchromb.2011.10.037 22100558

[30] Livak KJ Schmittgen TD . Analysis of relative gene expression data using real-time quantitative PCR and the 2(-Delta Delta C(T)) Method. Methods. (2001) 25:402–8. doi: 10.1006/meth.2001.1262 11846609

[31] Lei X Zhang S Barbour SE Bohrer A Ford EL Koizumi A . Spontaneous development of endoplasmic reticulum stress that can lead to diabetes mellitus is associated with higher calcium-independent phospholipase A2 expression: a role for regulation by SREBP-1. J Biol Chem. (2010) 285:6693–705. doi: 10.1074/jbc.M109.084293 20032468 PMC2825464

[32] Zhu X Gan-Schreier H Otto A-C Cheng Y Staffer S Tuma-Kellner S . iPla2β deficiency in mice fed with MCD diet does not correct the defect of phospholipid remodeling but attenuates hepatocellular injury via an inhibition of lipid uptake genes. Biochim Biophys Acta Mol Cell Biol Lipids. (2019) 1864:677–87. doi: 10.1016/j.bbalip.2019.02.003 30735854

[33] Su X Mancuso DJ Bickel PE Jenkins CM Gross RW . Small interfering RNA knockdown of calcium-independent phospholipases A2 beta or gamma inhibits the hormone-induced differentiation of 3T3-L1 preadipocytes. J Biol Chem. (2004) 279:21740–8. doi: 10.1074/jbc.M314166200 15024020

[34] Park P-H Sanz-Garcia C Nagy LE . Adiponectin as an anti-fibrotic and anti-inflammatory adipokine in the liver. Curr Pathobiol Rep. (2015) 3:243–52. doi: 10.1007/s40139-015-0094-y 26858914 PMC4743902

[35] Lauber K Bohn E Kröber SM Xiao Y-J Blumenthal SG Lindemann RK . Apoptotic cells induce migration of phagocytes via caspase-3-mediated release of a lipid attraction signal. Cell. (2003) 113:717–30. doi: 10.1016/s0092-8674(03)00422-7 12809603

[36] Di Gioia M Spreafico R Springstead JR Mendelson MM Joehanes R Levy D . Endogenous oxidized phospholipids reprogram cellular metabolism and boost hyperinflammation. Nat Immunol. (2020) 21:42–53. doi: 10.1038/s41590-019-0539-2 31768073 PMC6923570

[37] Mishra RS Carnevale KA Cathcart MK . iPLA2beta: front and center in human monocyte chemotaxis to MCP-1. J Exp Med. (2008) 205:347–59. doi: 10.1084/jem.20071243 18208975 PMC2271028

[38] Klement L Jansakun C Yan B Staffer S Tuma-Kellner S Altamura S . Myeloid-specific deletion of group VIA calcium-independent phospholipase A2 induces pro-inflammatory LPS response predominantly in male mice via MIP-1α activation. Biochim Biophys Acta Mol Basis Dis. (2024) 1870:167016. doi: 10.1016/j.bbadis.2024.167016 38198970

[39] Ward JM Anver MR Haines DC Benveniste RE . Chronic active hepatitis in mice caused by Helicobacter hepaticus. Am J Pathol. (1994) 145:959–68. doi: 10.1093/jnci/86.16.1222 7943185 PMC1887338

[40] Pekala PH Kawakami M Angus CW Lane MD Cerami A . Selective inhibition of synthesis of enzymes for de novo fatty acid biosynthesis by an endotoxin-induced mediator from exudate cells. Proc Natl Acad Sci USA. (1983) 80:2743–7. doi: 10.1073/pnas.80.9.2743 6133282 PMC393904

[41] Listenberger LL Han X Lewis SE Cases S Farese RV Ory DS . Triglyceride accumulation protects against fatty acid-induced lipotoxicity. Proc Natl Acad Sci USA. (2003) 100:3077–82. doi: 10.1073/pnas.0630588100 12629214 PMC152249

[42] Björkegren J Beigneux A Bergo MO Maher JJ Young SG . Blocking the secretion of hepatic very low density lipoproteins renders the liver more susceptible to toxin-induced injury. J Biol Chem. (2002) 277:5476–83. doi: 10.1074/jbc.M108514200 11739387

[43] Chakravarthy MV Pan Z Zhu Y Tordjman K Schneider JG Coleman T . New” hepatic fat activates PPARalpha to maintain glucose, lipid, and cholesterol homeostasis. Cell Metab. (2005) 1:309–22. doi: 10.1016/j.cmet.2005.04.002 16054078

[44] Sehayek E Wang R Ono JG Zinchuk VS Duncan EM Shefer S . Localization of the PE methylation pathway and SR-BI to the canalicular membrane: evidence for apical PC biosynthesis that may promote biliary excretion of phospholipid and cholesterol. J Lipid Res. (2003) 44:1605–13. doi: 10.1194/jlr.M200488-JLR200 12810817

[45] Aly A Carlson K Johansson C Kirstein P Rössner S Wallentin L . Lipoprotein abnormalities in patients with early primary biliary cirrhosis. Eur J Clin Invest. (1984) 14:155–62. doi: 10.1111/j.1365-2362.1984.tb02106.x 6428906

[46] Tietge UJ Boker KH Bahr MJ Weinberg S Pichlmayr R Schmidt HH . Lipid parameters predicting liver function in patients with cirrhosis and after liver transplantation. Hepatogastroenterology. (1998) 45:2255–60. 9951906

[47] Stelmanska E Swierczynski J . Up-regulation of lipogenic enzyme genes expression in inguinal white adipose tissue of female rats by progesterone. J Steroid Biochem Mol Biol. (2013) 134:37–44. doi: 10.1016/j.jsbmb.2012.10.006 23079166

[48] Jeong KJ Mukae M Lee SR Kim S-Y Kim SH Cho Y-E . Progesterone increases hepatic lipid content and plasma lipid levels through PR- B-mediated lipogenesis. BioMed Pharmacother. (2024) 172:116281. doi: 10.1016/j.biopha.2024.116281 38364736

[49] Xu F Jansakun C Li G Biswas U Poschet G Staffer S . Myeloid-specific deficiency of group VIA calcium-independent phospholipase A2 preconditions myeloid cells for injury resolution after acetaminophen exposure. BioMed Pharmacother. (2025) 187:118146. doi: 10.1016/j.biopha.2025.118146 40344700

[50] LaFrance M Hansel W . Role of arachidonic acid and its metabolites in the regulation of progesterone and oxytocin release from the bovine corpus luteum. Proc Soc Exp Biol Med. (1992) 201:106–13. doi: 10.3181/00379727-201-43487 1528904

[51] McFadden N Bailey D Carrara G Benson A Chaudhry Y Shortland A . Norovirus regulation of the innate immune response and apoptosis occurs via the product of the alternative open reading frame 4. PloS Pathog. (2011) 7:e1002413. doi: 10.1371/journal.ppat.1002413 22174679 PMC3234229

[52] Itagaki T Shimizu I Cheng X Yuan Y Oshio A Tamaki K . Opposing effects of oestradiol and progesterone on intracellular pathways and activation processes in the oxidative stress induced activation of cultured rat hepatic stellate cells. Gut. (2005) 54:1782–9. doi: 10.1136/gut.2005.053278 16284289 PMC1774809

[53] Toyoda Y Endo S Tsuneyama K Miyashita T Yano A Fukami T . Mechanism of exacerbative effect of progesterone on drug-induced liver injury. Toxicol Sci. (2012) 126:16–27. doi: 10.1093/toxsci/kfr326 22157104

[54] Ryan VH Primiani CT Rao JS Ahn K Rapoport SI Blanchard H . Coordination of gene expression of arachidonic and docosahexaenoic acid cascade enzymes during human brain development and aging. PloS One. (2014) 9:e100858. doi: 10.1371/journal.pone.0100858 24963629 PMC4070994

[55] Gao PY Ou YN Wang HF Wang ZB Fu Y He XY . Associations of liver dysfunction with incident dementia, cognition, and brain structure: a prospective cohort study of 431699 adults. J Neurochem. (2024) 168:26–38. doi: 10.1111/jnc.15988 37830502

[56] Silvey S Sterling RK French E Godschalk M Gentili A Patel N . A possible reversible cause of cognitive impairment: undiagnosed cirrhosis and potential hepatic encephalopathy in patients with dementia. Am J Med. (2024) 137:1082–1087.e1. doi: 10.1016/j.amjmed.2024.06.014 38942345 PMC11513228

[57] Wilkins WP Barbour SE . Group VI phospholipases A2: homeostatic phospholipases with significant potential as targets for novel therapeutics. Curr Drug Targets. (2008) 9:683–97. doi: 10.2174/138945008785132385 18691015

