# Supplementary material for: iPLA2β-null mice exposed to natural pathogens exhibit hepatocellular fibrotic injury with male-biased alteration of glycerolipid metabolism
Source: Front Immunol. 2026 Jul 8;17:1839554. doi: 10.3389/fimmu.2026.1839554 (PMC13388193; doi:10.3389/fimmu.2026.1839554)
Supplement: Supplementary file 1 [file DataSheet1.pdf]

IBF
Universität Heidelberg
INF 347

Gesundheitskontrolle

Maus

Lab.Nr.:	1063-1064	Raum:	2.06	Unit:	KEB
				Probennahme:	02.12.2014

		Positiv	Proben	Methode
	**Viren**			
1	Mouse hepatitis virus (MHV)	0	2	IFA
2	Reovirus type 3			IFA
3	Theiler (TMEV)	0	2	IFA
4	Pneumonia virus of mice (PVM)			IFA
5	Sendai virus			IFA
6	Minute virus of mice (MVM)	0	2	IFA
7	Mouse parvovirus (MPV)	0	2	IFA
8	Ectromelia virus			IFA
9	Lymphocytic choriomeningitis virus (LCM)			IFA
10	Adeno (MAd K87)			IFA
11	Adeno (MAd FL)			IFA
12	Hantaanvirus	0	2	IFA
13	Mouse rota virus (EDIM)	0	2	IFA
14	Norovirus	2	2	IFA (ELISA)
	**Bakterien**			
1	Mycoplasma pulmonis			IFA
2	Clostridium piliforme (Tyzzer)			IFA
3	Pasteurella pneumotropica			CULT
4	Bordetella bronchiseptica			CULT
5	Citrobacter rodentium			CULT
6	Corynebacterium kutscheri			CULT
7	Corynebacterium bovis			CULT
8	Klebsiella pneumoniae, oxytoca			CULT
9	Pasteurella multocida			CULT
10	Pasteurella spp			CULT
11	Proteus mirabilis et vulgaris			CULT
12	Pseudomonas aeruginosa			CULT
13	Salmonella spp			CULT
14	Staphylococcus aureus			CULT
15	Streptococcus ß häm. (andere als Gruppe D)			CULT
16	Streptococcus pneumoniae			CULT
17	Yersinia pseudotuberculosis			CULT
18	Helicobacter sp.			PCR
19	Helicobacter bilis			PCR
20	Helicobacter hepaticus			PCR
21	Helicobacter typhlonius			PCR
22	Streptobacillus moniliformis			PCR
	**Parasiten**			
1	Ektoparasiten			MICR
2	Protozoen			MICR
3	Helminthen			MICR
	Pathologische Veränderungen			

IBF
Universität Heidelberg
INF 347

Gesundheitskontrolle

Maus

Lab.Nr.	942-943	Raum:	2.06	Unit:	KEB
				Probennahme:	21.10.2014

		Positiv	Proben	Methode
	**Viren**			
1	Mouse hepatitis virus (MHV)	0	2	IFA
2	Reovirus type 3			IFA
3	Theiler (TMEV)	0	2	IFA
4	Pneumonia virus of mice (PVM)			IFA
5	Sendai virus			IFA
6	Minute virus of mice (MVM)	0	2	IFA
7	Mouse parvovirus (MPV)	0	2	IFA
8	Ectromelia virus			IFA
9	Lymphocytic choriomeningitis virus (LCM)			IFA
10	Adeno (MAd K87)			IFA
11	Adeno (MAd FL)			IFA
12	Hantaanvirus	0	2	IFA
13	Mouse rota virus (EDIM)	0	2	IFA
14	Norovirus	0	2	IFA (ELISA)
	**Bakterien**			
1	Mycoplasma pulmonis			IFA
2	Clostridium piliforme (Tyzzer)			IFA
3	Pasteurella pneumotropica	0	2	CULT
4	Bordetella bronchiseptica	0	2	CULT
5	Citrobacter rodentium	0	2	CULT
6	Corynebacterium kutscheri	0	2	CULT
7	Corynebacterium bovis	0	2	CULT
8	Klebsiella pneumoniae, oxytoca	0	2	CULT
9	Pasteurella multocida	0	2	CULT
10	Pasteurella spp	0	2	CULT
11	Proteus mirabilis et vulgaris	0	2	CULT
12	Pseudomonas aeruginosa	0	2	CULT
13	Salmonella spp	0	2	CULT
14	Staphylococcus aureus	0	2	CULT
15	Streptococcus ß häm. (andere als Gruppe D)	0	2	CULT
16	Streptococcus pneumoniae	0	2	CULT
17	Yersinia pseudotuberculosis	0	2	CULT
18	Helicobacter sp.	2	2	PCR
19	Helicobacter bilis	0	2	PCR
20	Helicobacter hepaticus	2	2	PCR
21	Helicobacter typhlonius	2	2	PCR
22	Streptobacillus moniliformis	0	2	PCR
	**Parasiten**			
1	Ektoparasiten	0	2	MICR
2	Protozoen	2	2	MICR
3	Helminthen	0	2	MICR
	Pathologische Veränderungen			

√

IBF
Universität Heidelberg
INF 347

Gesundheitskontrolle

Maus

Lab.Nr.:	859/ 860	Raum:	2.06	Unit:	KEB
				Probennahme:	02.09.2014

		Positiv	Proben	Methode
	**Viren**			
1	Mouse hepatitis virus (MHV)	0	2	IFA
2	Reovirus type 3			IFA
3	Theiler (TMEV)	0	2	IFA
4	Pneumonia virus of mice (PVM)			IFA
5	Sendai virus			IFA
6	Minute virus of mice (MVM)	0	2	IFA
7	Mouse parvovirus (MPV)	0	2	IFA
8	Ectromelia virus			IFA
9	Lymphocytic choriomeningitis virus (LCM)			IFA
10	Adeno (MAd K87)			IFA
11	Adeno (MAd FL)			IFA
12	Hantaanvirus	0	2	IFA
13	Mouse rota virus (EDIM)	0	2	IFA
14	Norovirus	2	2	IFA (ELISA)
	**Bakterien**			
1	Mycoplasma pulmonis			IFA
2	Clostridium piliforme (Tyzzer)			IFA
3	Pasteurella pneumotropica			CULT
4	Bordetella bronchiseptica			CULT
5	Citrobacter rodentium			CULT
6	Corynebacterium kutscheri			CULT
7	Corynebacterium bovis			CULT
8	Klebsiella pneumoniae, oxytoca			CULT
9	Pasteurella multocida			CULT
10	Pasteurella spp			CULT
11	Proteus mirabilis et vulgaris			CULT
12	Pseudomonas aeruginosa			CULT
13	Salmonella spp			CULT
14	Staphylococcus aureus			CULT
15	Streptococcus ß häm. (andere als Gruppe D)			CULT
16	Streptococcus pneumoniae			CULT
17	Yersinia pseudotuberculosis			CULT
18	Helicobacter sp.			PCR
19	Helicobacter bilis			PCR
20	Helicobacter hepaticus			PCR
21	Helicobacter typhlonius			PCR
22	Streptobacillus moniliformis			PCR
	**Parasiten**			
1	Ektoparasiten			MICR
2	Protozoen			MICR
3	Helminthen			MICR
	Pathologische Veränderungen			

IBF
Universität Heidelberg
INF 347

Gesundheitskontrolle

Maus

Lab.Nr.	741 - 742	Raum:	2.06	Unit:	KEB
				Probennahme:	22.07.2014

		Positiv	Proben	Methode
	**Viren**			
1	Mouse hepatitis virus (MHV)	0	2	IFA
2	Reovirus type 3			IFA
3	Theiler (TMEV)	0	2	IFA
4	Pneumonia virus of mice (PVM)			IFA
5	Sendai virus			IFA
6	Minute virus of mice (MVM)	0	2	IFA
7	Mouse parvovirus (MPV)	0	2	IFA
8	Ectromelia virus			IFA
9	Lymphocytic choriomeningitis virus (LCM)			IFA
10	Adeno (MAd K87)			IFA
11	Adeno (MAd FL)			IFA
12	Mouse thymic virus (MTV)			IFA
13	Mouse polyomavirus			IFA
14	Hantaanvirus	0	2	IFA
15	Mouse cytomegali virus (MCMV)			IFA
16	Mouse rota virus (EDIM)	0	2	IFA
17	Mouse K virus			ELISA
18	Norovirus	2	2	IFA (ELISA)
19	Lactic dehydrogenase virus (LDV)			enzym.
	**Bakterien**			
1	CAR Bacillus			ELISA
2	Mycoplasma pulmonis			IFA
3	Clostridium piliforme (Tyzzer)			IFA
4	Pasteurella pneumotropica	0	2	CULT
5	Bordetella bronchiseptica	0	2	CULT
6	Citrobacter rodentium	0	2	CULT
7	Corynebacterium kutscheri	0	2	CULT
8	Corynebacterium bovis	0	2	CULT
9	Klebsiella pneumoniae, oxytoca	0	2	CULT
10	Pasteurella multocida	0	2	CULT
11	Pasteurella spp	0	2	CULT
12	Proteus mirabilis et vulgaris	0	2	CULT
13	Pseudomonas aeruginosa	0	2	CULT
14	Salmonella spp	0	2	CULT
15	Staphylococcus aureus	0	2	CULT
16	Streptococcus ß häm. (andere als Gruppe D)	0	2	CULT
17	Streptococcus pneumoniae	0	2	CULT
18	Yersinia pseudotuberculosis	0	2	CULT
19	Helicobacter sp.	2	2	PCR
20	Helicobacter bilis	0	2	PCR
21	Helicobacter hepaticus	2	2	PCR
22	Helicobacter typhlonius	2	2	PCR
23	Streptobacillus moniliformis	0	2	PCR
	**Parasiten**			
1	Ektoparasiten	0	2	MICR
2	Protozoen	0	2	MICR
3	Helminthen	0	2	MICR
	Pathologische Veränderungen			

✓ Bn

IBF
Universität Heidelberg
INF 347

Gesundheitskontrolle

Maus

Lab.Nr.	591 - 592	Raum:	2.06	Unit:	KEB
				Probennahme:	10.06.2014

		Positiv	Proben	Methode
	**Viren**			
1	Mouse hepatitis virus (MHV)	0	2	IFA
2	Reovirus type 3			IFA
3	Theiler (TMEV)	0	2	IFA
4	Pneumonia virus of mice (PVM)			IFA
5	Sendai virus			IFA
6	Minute virus of mice (MVM)	0	2	IFA
7	Mouse parvovirus (MPV)	0	2	IFA
8	Ectromelia virus			IFA
9	Lymphocytic choriomeningitis virus (LCM)			IFA
10	Adeno (MAd K87)			IFA
11	Adeno (MAd FL)			IFA
12	Mouse thymic virus (MTV)			IFA
13	Mouse polyomavirus			IFA
14	Hantaanvirus	0	2	IFA
15	Mouse cytomegali virus (MCMV)			IFA
16	Mouse rota virus (EDIM)	0	2	IFA
17	Mouse K virus			ELISA
18	Norovirus	2	2	IFA (ELISA)
19	Lactic dehydrogenase virus (LDV)			enzym.
	**Bakterien**			
1	CAR Bacillus			ELISA
2	Mycoplasma pulmonis			IFA
3	Clostridium piliforme (Tyzzer)			IFA
4	Pasteurella pneumotropica	0	2	IFA
5	Bordetella bronchiseptica			CULT
6	Citrobacter rodentium			CULT
7	Corynebacterium kutscheri			CULT
8	Corynebacterium bovis			CULT
9	Klebsiella oxytoca			CULT
10	Klebsiella pneumoniae			CULT
11	Pasteurella spp			CULT
12	Proteus mirabilis et vulgaris			CULT
13	Pseudomonas aeruginosa			CULT
14	Salmonella spp			CULT
15	Staphylococcus aureus			CULT
16	Streptococcus ß häm. (andere als Gruppe D)			CULT
17	Streptococcus pneumoniae			CULT
18	Yersinia pseudotuberculosis			CULT
19	Helicobacter sp.			PCR
20	Helicobacter bilis			PCR
21	Helicobacter hepaticus			PCR
22	Helicobacter typhlonius			PCR
23	Streptobacillus moniliformis			PCR
	**Parasiten**			
1	Ektoparasiten			MICR
2	Protozoen			MICR
3	Helminthen			MICR
	Pathologische Veränderungen			

IBF
Universität Heidelberg
INF 347

Gesundheitskontrolle

Maus

Lab.Nr.	424 - 425	Raum:	2.06	Unit:	KEB
				Probennahme:	22.04.2014

		Positiv	Proben	Methode
	**Viren**			
1	Mouse hepatitis virus (MHV)	0	2	IFA
2	Reovirus type 3			IFA
3	Theiler (TMEV)	0	2	IFA
4	Pneumonia virus of mice (PVM)			IFA
5	Sendai virus			IFA
6	Minute virus of mice (MVM)	0	2	IFA
7	Mouse parvovirus (MPV)	0	2	IFA
8	Ectromelia virus			IFA
9	Lymphocytic choriomeningitis virus (LCM)			IFA
10	Adeno (MAd K87)			IFA
11	Adeno (MAd FL)			IFA
12	Mouse thymic virus (MTV)			IFA
13	Mouse polyomavirus			IFA
14	Hantaanvirus	0	2	IFA
15	Mouse cytomegali virus (MCMV)			IFA
16	Mouse rota virus (EDIM)	0	2	IFA
17	Mouse K virus			ELISA
18	Norovirus	0	2	IFA (ELISA)
19	Lactic dehydrogenase virus (LDV)			enzym.
	**Bakterien**			
1	CAR Bacillus			ELISA
2	Mycoplasma pulmonis			IFA
3	Clostridium piliforme (Tyzzer)			IFA
4	Pasteurella pneumotropica	0	2	IFA
5	Bordetella bronchiseptica	0	2	CULT
6	Citrobacter rodentium	0	2	CULT
7	Corynebacterium kutscheri	0	2	CULT
8	Corynebacterium bovis	0	2	CULT
9	Klebsiella pneumoniae, oxytoca	0	2	CULT
10	Pasteurella multocida	0	2	CULT
11	Pasteurella spp	0	2	CULT
12	Proteus mirabilis et vulgaris	0	2	CULT
13	Pseudomonas aeruginosa	0	2	CULT
14	Salmonella spp	0	2	CULT
15	Staphylococcus aureus	0	2	CULT
16	Streptococcus ß häm. (andere als Gruppe D)	0	2	CULT
17	Streptococcus pneumoniae	0	2	CULT
18	Yersinia pseudotuberculosis	0	2	CULT
19	Helicobacter sp.	2	2	PCR
20	Helicobacter bilis	0	2	PCR
21	Helicobacter hepaticus	2	2	PCR
22	Helicobacter typhlonius	0	2	PCR
23	Streptobacillus moniliformis	0	2	PCR
	**Parasiten**			
1	Ektoparasiten	0	2	MICR
2	Protozoen	0	2	MICR
3	Helminthen	0	2	MICR
	Pathologische Veränderungen			

✓

IBF
Universität Heidelberg
INF 347

Gesundheitskontrolle

Maus

Lab.Nr.	293-294	Raum:	2.06	Unit:	KEB
				Probennahme:	11.03.2014

		Positiv	Proben	Methode
	Viren			
1	Mouse hepatitis virus (MHV)	0	2	IFA
2	Reovirus type 3	0	2	IFA
3	Theiler (TMEV)	0	2	IFA
4	Pneumonia virus of mice (PVM)	0	2	IFA
5	Sendai virus	0	2	IFA
6	Minute virus of mice (MVM) / Mouse parvovirus (MPV)	0	2	IFA
7	Ectromelia virus	0	2	IFA
8	Lymphocytic choriomeningitis virus (LCM)	0	2	IFA
9	Adeno (MAd K87)	0	2	IFA
10	Adeno (MAd FL)	0	2	IFA
11	Mouse thymic virus (MTV)	0	2	IFA
12	Mouse cytomegali virus (MCMV)	0	2	IFA
13	Mouse rota virus (EDIM)	0	2	IFA
14	Lactic dehydrogenase virus (LDV)			enzym.
15	Norovirus	0	2	IFA
16	Hantaanvirus	0	2	IFA
17	Mouse K virus	0	2	IFA
18	Mouse polyomavirus	0	2	IFA
	Bakterien			
1	Citrobacter rodentium			CULT
2	Clostridium piliforme (Tyzzer)			MICR
2a	Clostridium piliforme (Tyzzer)	0	2	IFA
3	Corynebacterium kutscheri			CULT
4	Mycoplasma pulmonis	0	2	IFA
5	Pasteurella pneumotropica	0	2	IFA
6	Pasteurella spp			CULT
7	Salmonella spp			CULT
8	Streptococcus ß häm.			CULT
9	Streptococcus pneumoniae			CULT
10	Staphylococcus aureus/intermedius			CULT
11	Proteus mirabilis et vulgaris			CULT
12	Klebsiella pneumoniae, oxytoca			CULT
13	Pseudomonas aeruginosa			CULT
14	Bordetella bronchiseptica			CULT
15	Corynebacterium bovis			CULT
2	Yersinia pseudotuberculosis			CULT
17	CAR Bacillus	0	2	IFA
18	Helicobacter sp.			PCR
19	Helicobacter bilis			PCR
20	Helicobacter hepaticus			PCR
21	Helicobacter typhlonius			PCR
22	Streptobacillus moniliformis			CULT
	Parasiten			
1	Ektoparasiten			MICR
2	Protozoen			MICR
3	Helminthen			MICR
	Pathologische Veränderungen			

IBF
Universität Heidelberg
INF 347

Gesundheitskontrolle

Maus

Lab.Nr.	145-146	Raum:	2.06	Unit:	KEB
				Probennahme:	28.01.2014

		Positiv	Proben	Methode
	Viren			
1	Mouse hepatitis virus (MHV)	0	2	IFA
2	Reovirus type 3	0	2	IFA
3	Theiler (TMEV)	0	2	IFA
4	Pneumonia virus of mice (PVM)	0	2	IFA
5	Sendai virus	0	2	IFA
6	Minute virus of mice (MVM) / Mouse parvovirus (MPV)	0	2	IFA
7	Ectromelia virus	0	2	IFA
8	Lymphocytic choriomeningitis virus (LCM)	0	2	IFA
9	Adeno (MAd K87)	0	2	IFA
10	Adeno (MAd FL)	0	2	IFA
11	Mouse thymic virus (MTV)	0	2	IFA
12	Mouse cytomegali virus (MCMV)	0	2	IFA
13	Mouse rota virus (EDIM)	0	2	IFA
14	Lactic dehydrogenase virus (LDV)	0	2	enzym.
15	Norovirus	0	2	IFA
16	Hantaanvirus	0	2	IFA
17	Mouse K virus	0	2	IFA
18	Mouse polyomavirus	0	2	IFA
	Bakterien			
1	Citrobacter rodentium	0	2	CULT
2	Clostridium piliforme (Tyzzer)	0	2	MICR
2a	Clostridium piliforme (Tyzzer)	0	2	IFA
3	Corynebacterium kutscheri	0	2	CULT
4	Mycoplasma pulmonis	0	2	IFA
5	Pasteurella pneumotropica	0	2	IFA
6	Pasteurella spp	0	2	CULT
7	Salmonella spp	0	2	CULT
8	Streptococcus ß häm.	0	2	CULT
9	Streptococcus pneumoniae	0	2	CULT
10	Staphylococcus aureus/intermedius	0	2	CULT
11	Proteus mirabilis et vulgaris	0	2	CULT
12	Klebsiella pneumoniae, oxytoca	0	2	CULT
13	Pseudomonas aeruginosa	0	2	CULT
14	Bordetella bronchiseptica	0	2	CULT
15	Corynebacterium bovis	0	2	CULT
2	Yersinia pseudotuberculosis	0	2	CULT
17	CAR Bacillus	0	2	IFA
18	Helicobacter sp.	2	2	PCR
19	Helicobacter bilis	0	2	PCR
20	Helicobacter hepaticus	2	2	PCR
21	Helicobacter typhlonius	0	2	PCR
22	Streptobacillus moniliformis	0	2	CULT
	Parasiten			
1	Ektoparasiten	0	2	MICR
2	Protozoen	0	2	MICR
3	Helminthen	0	2	MICR
	Pathologische Veränderungen			
